# Image based background magnetic field correction for aortic and pulmonary artery flow measurement using phase contrast

**DOI:** 10.1186/1532-429X-13-S1-P355

**Published:** 2011-02-02

**Authors:** Joshua Y Cheng

**Affiliations:** 1 St. Francis Hospital The Heart Center, Roslyn, NY, USA

## Objectives

We investigated background phase corrections for accurate aortic and pulmonary artery (PA) flow assessment alternative to additional static fluid phantom calibration.

## Background

Background phase shift due to magnetic field inhomogeneity can cause significant error in aortic and PA flow measurements.

## Methods

Twelve volunteers underwent breath-hold through-plane phase contrast (PC) imaging of cross sectional view of the ascending aorta and PA. PC imaging was immediately repeated on a static fluid-filled phantom. Phase images were analyzed using QFlow (Medis, Leiden, Netherlands.). Phase shift was also analyzed in static background and in surrounding tissues including chest wall fat and muscle, lung and vertebral body. For PA flow Images only lung and static background were consistently present. Since background phase shift is linearly decreased from anterior to posterior, we calculated background phase shift at the aortic position using linear regression against distance of the aorta from anterior chest wall. Given the proximity of the aortic and PA in z direction the calculated aortic background phase was applied to the PA as well.

## Result

The average aorta flow velocity was 11.2±3.5 cm/s ranging 5.9-16.7 cm/s. The measured phantom phase velocity was 0.8±0.5 cm/s accounting for 8.1±5.8% of aortic phase. The aortic phase change comparing phase corrected by bone, muscle, fat, lung and static background to phase corrected by phantom were 10.8±13.3%, -1.5±9.6%, -6.3±11.8%, 15.5±28.8 and -17.6±20.6%, respectively. The calculated background phase in aorta position was 0.9±0.4 cm/s which was not significant different from measured phase in the phantom (p=0.199). The average PA flow velocity was 11.8±2.6 cm/s and the measured phantom phase velocity in PA position was 0.7±0.3 cm/s accounting for 6.6±3.8% of the PA phase. The PA phase change comparing phase corrected by lung and static background to phase corrected by phantom were 8.4±26.8% and 6.0±7.7%. In contrast, the calculated background phase was not significantly different from measured phase in the phantom in PA position (p=0.239).

## Conclusion

Background phase corrections using surrounding tissues and static background were not suitable for clinical use because of large variations. However, the calculated background phase might be a promising alternative to phantom calibration.

